# Supplementary material for: Input-dependent regulation of excitability controls dendritic maturation in somatosensory thalamocortical neurons
Source: Nat Commun. 2017 Dec 8;8:2015. doi: 10.1038/s41467-017-02172-1 (PMC5722950; doi:10.1038/s41467-017-02172-1)
Supplement: Supplementary file 1 — Supplementary Information [file 41467_2017_2172_MOESM1_ESM.pdf]

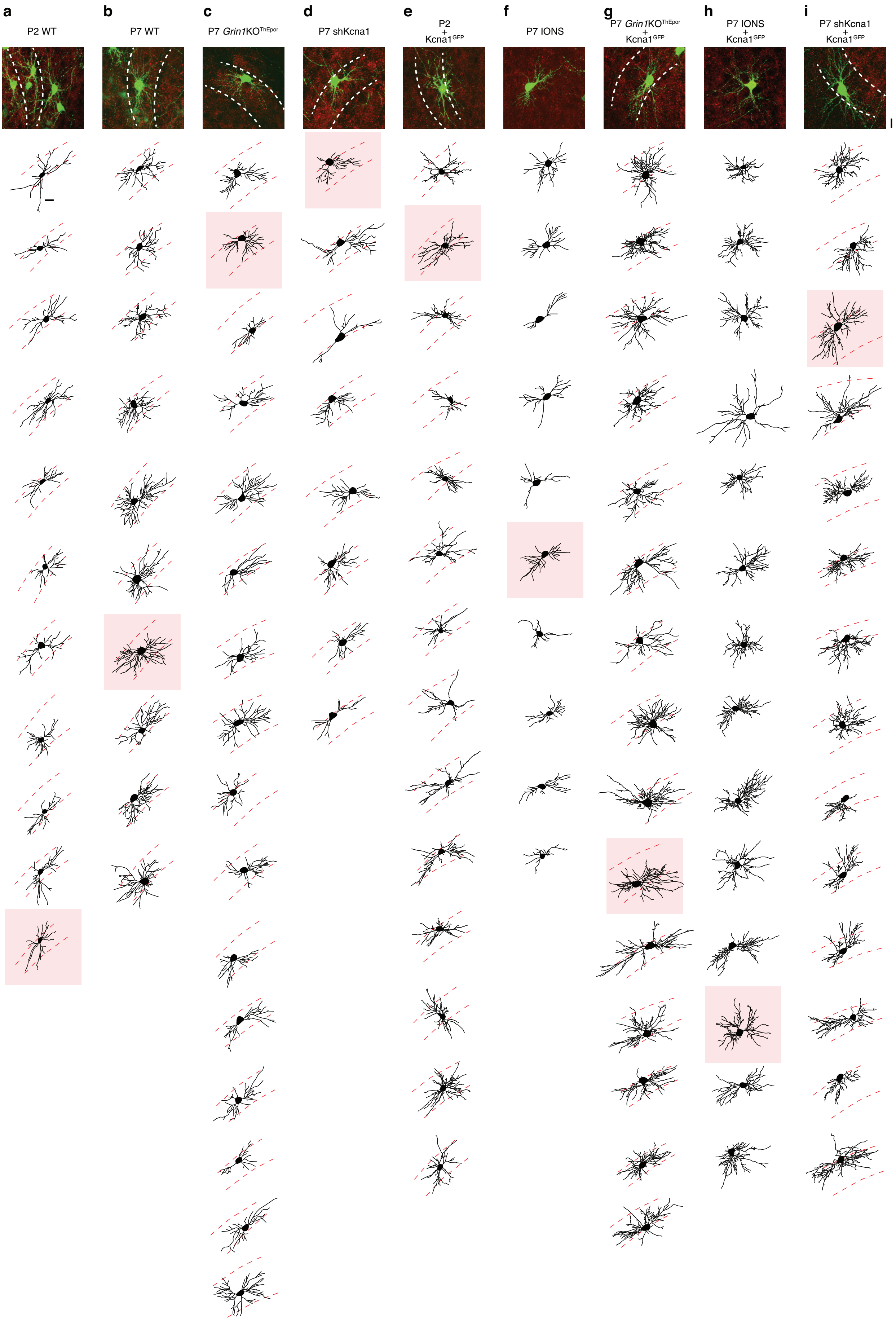

Supplementary Figure 1

Reconstruction of the VPM neurons used for dendritic analyses in this study.

Photomicrograph example of GFP⁺ neurons in the VPM and reconstruction of the VPM neurons for each conditions (pink background highlight reconstruted neurons shown on photomicrograph, red dotted lines delineate barreloid). Scale bars: 20 µm.

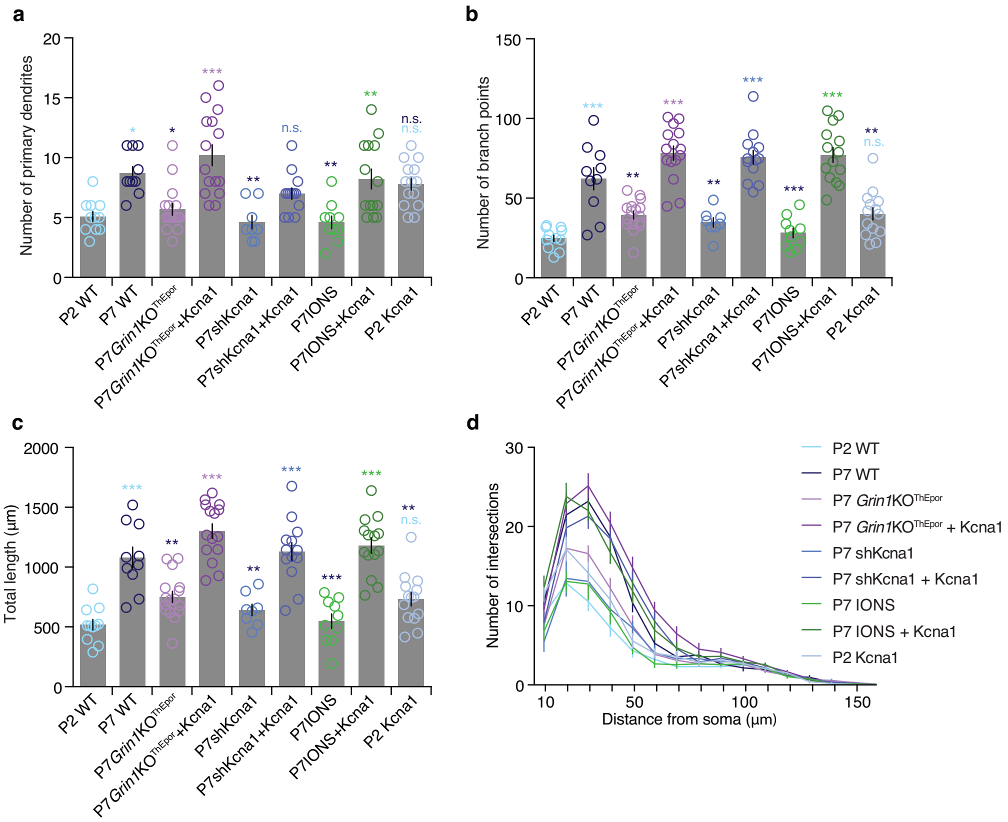

Supplementary Figure 2

Quantification of the dendritic complexity of VPM neurons for each experimental condition.

(**a**) Quantification of the number of primary dendrites for each experimental condition. One-way ANOVA with Tukey post-hoc test; * $P < 0.05$, ** $P < 0.01$, *** $P < 0.001$, n.s., not significant. (P2 WT (n = 11 cells from 2 mice): 5.09 ± 0.41; P7 WT (n = 10 cells from 2 mice): 8.7 ± 0.56; P7 *Grin1*KO^{ThEpor} (n = 16 cells from 3 mice): 5.68 ± 0.48; P7 *Grin1*KO^{ThEpor} + Kcna1 (n = 15 cells from 6 mice): 10.2 ± 0.88; P7 shKcna1 (n = 8 cells from 2 mice): 4.62 ± 0.56; P7 shKcna1 + Kcna1 (n = 13 cells from 4 mice): 7 ± 0.46; P7 IONS (n = 10 cells from 2 mice): 4.6 ± 0.52; P7 IONS + Kcna1 (n = 14 cells from 7 mice): 8.21 ± 0.82; P2 Kcna1 (n = 14 cells from 4 mice): 7.78 ± 0.50.) (**b**) Quantification of the number of branch points for each experimental condition. One-way ANOVA with Tukey post-hoc test; * $P < 0.05$, ** $P < 0.01$, *** $P < 0.001$, n.s., not significant. (P2 WT (n = 11 cells from 2 mice): 25 ± 2.03; P7 WT (n = 10 cells from 2 mice): 62.4 ± 6.98; P7 *Grin1*KO^{ThEpor} (n = 16 cells from 3 mice): 39.56 ± 2.38; P7 *Grin1*KO^{ThEpor} + Kcna1 (n = 15 cells from 6 mice): 78.47 ± 4.42; P7 shKcna1 (n = 8 cells from 2 mice): 35 ± 2.88; P7 shKcna1 + Kcna1 (n = 13 cells from 4 mice): 75.85 ± 4.32; P7 IONS (n = 10 cells from 2 mice): 28.4 ± 3.08; P7 IONS + Kcna1 (n = 14 cells from 7 mice): 77.36 ± 4.66; P2 Kcna1 (n = 14 cells from 4 mice): 40.14 ± 3.87.) (**c**) Quantification of the number of the total dendritic length for each experimental condition. One-way ANOVA with Tukey post-hoc test; * $P < 0.05$, ** $P < 0.01$, *** $P < 0.001$, n.s., not significant. (P2 WT (n = 11 cells from 2 mice): 516.9 ± 44.97; P7 WT (n = 10 cells from 2 mice): 1076 ± 87.65; P7 *Grin1*KO^{ThEpor} (n = 16 cells from 3 mice): 749.3 ± 46.58; P7 *Grin1*KO^{ThEpor} + Kcna1 (n = 15 cells from 6 mice): 1297 ± 60.93; P7 shKcna1 (n = 8 cells from 2 mice): 641.1 ± 47.47; P7 shKcna1 + Kcna1 (n = 13 cells from 4 mice): 1126 ± 74.27; P7 IONS (n = 10 cells from 2 mice): 547.5 ± 59.58; P7 IONS + Kcna1 (n = 14 cells from 7 mice): 1176 ± 64.24; P2 Kcna1 (n = 14 cells from 4 mice): 731.1 ± 56.66.) (**d**) Sholl analysis for each experimental condition. Data are expressed as mean ± S.E.M.

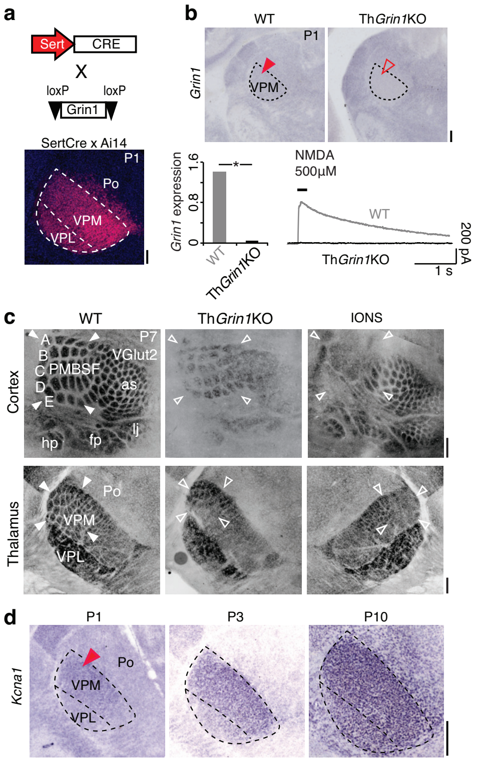

Supplementary Figure 3

Genetic deletion of Grin1 in VPM neurons impairs the formation of whisker-specific patterns.

(**a**) Top: Th*Grin1*KO mice were obtained by SertCre-mediated recombination of the *Grin1* floxed alleles. Bottom: Coronal section of Red Fluorescent Protein (RFP) expression driven by SertCre showing the specific recombination in the VPM. Scale bar: 100 µm. (**b**) Top: *In situ* hybridization for *Grin1* in coronal section of P1 brains (empty arrow shows the lack of *Grin1* in the VPM). Bottom left: qPCR analysis of *Grin1* mRNA expression in the VPM neurons of control and Th*Grin1*KO mice. Student's *t* test; * $P < 0.05$. Bottom right: Currents evoked by NMDA (500 µM) puff application. Absence of response reflects the lack of NMDAR-mediated excitation in VPM of Th*Grin1*KO. Scale bar: 200 µm. (**c**) Flattened cortex and coronal thalamic sections of P7 brains stained for VGlut2 reveal that whisker-related patterns fail to develop in Th*Grin1*KO mice as well as after infraorbital nerve section. Scale bar: 200 µm. (**d**) *In situ* hybridization for *Kcna1* showing its developmental expression. Scale bar: 250 µm.

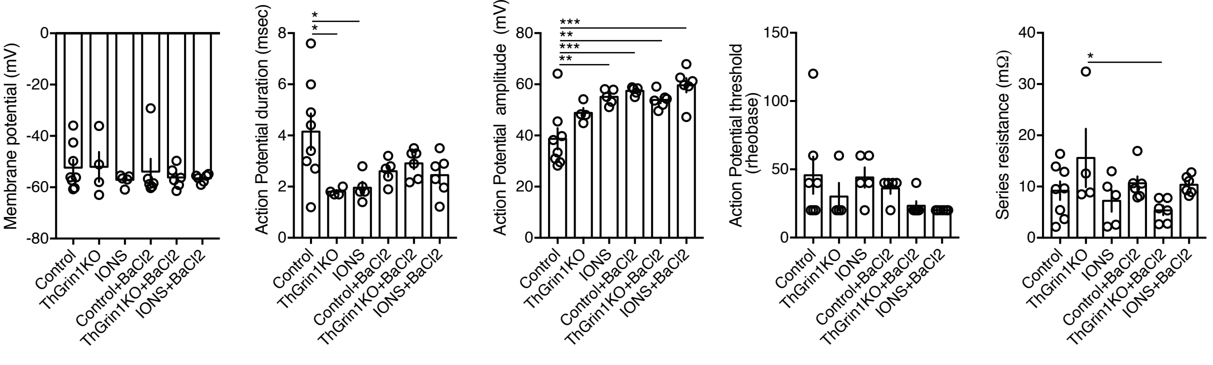

Supplementary Figure 4

Analysis of electrophysiological parameters at P15.

Graphical representation of the different electrophysiological parameters measured in Supplementary Table 2. One-way ANOVA with Tukey post-hoc test, * $P < 0.05$, ** $P < 0.01$, *** $P < 0.001$.

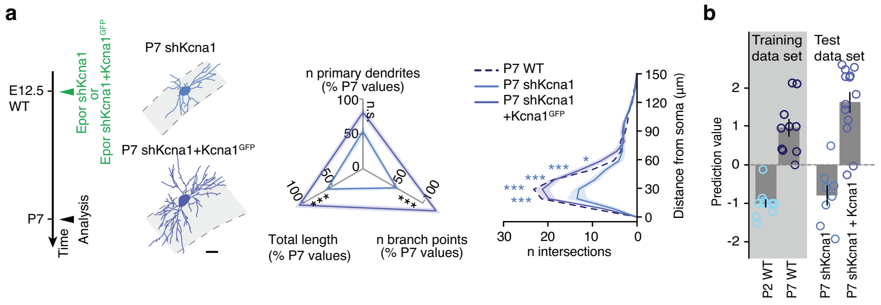

Supplementary Figure 5

***Kcna1* is important for the normal development of VPM neurons.**

(**a**) Overexpression of the human *Kcna1* rescues VPM neurons that were electroporated with shKcna1 (P7 shKcna1+Kcna1^{GFP} n = 14, from 4 mice). P7 WT data reported from Fig. 1b for the Sholl analysis. One-way ANOVA with Tukey post-hoc test; * $P < 0.05$, *** $P < 0.001$, n.s., not significant. Scale bar: 20 µm. (**b**) Linear model using dendritic complexity characteristics of P2 and P7 control VPM neurons as training sets. Dendritic complexity is restored back to control levels following human *Kcna1* overexpression.

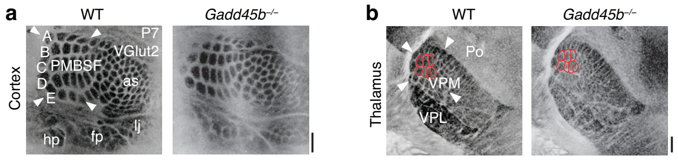

Supplementary Figure 6

Genetic deletion of Gadd45b does not detectably affect on the formation of whisker-specific patterns.

Flattened cortex (**a**) and thalamic coronal section (**b**) of P7 brains stained for VGlut2 reveal that whisker-related patterns develop normaly in *Gadd45b*$^{-/-}$ mice. Scale bars: 200 µm.

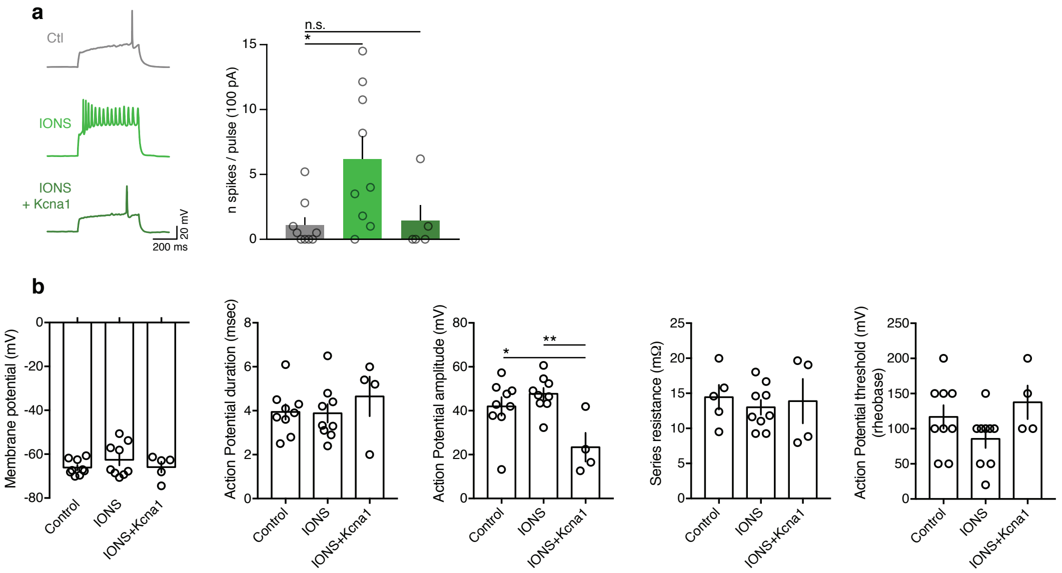

Supplementary Figure 7

Neuronal hyperexcitability is already present at P7 after IONS.

(**a**) At P7, IONS already leads to increased excitability of VPM neurons, which is rescued by Kcna1 overexpression (Ctl $n = 9$ cells from 4 mice, IONS $n = 9$ cells from 2 mice, IONS+Kcna1 $n = 5$ cells from 2 mice). One-way ANOVA with Tukey post-hoc test, * $P < 0.05$, n.s.: not significant. (**b**) Graphical representation of the different electrophysiological parameters measured in Supplementary Table 3. One-way ANOVA with Tukey post-hoc test, * $P < 0.05$, ** $P < 0.01$.

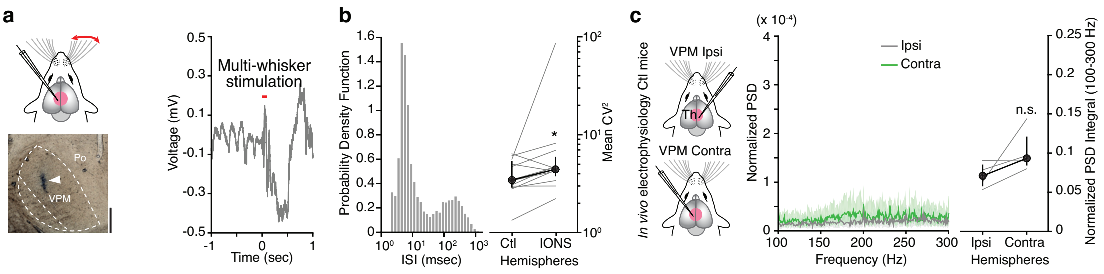

Supplementary Figure 8

Effect of IONS on VPM neurons *in vivo*.

(**a**) Example of an evoked local field potential (LFP) response from a control VPM after multi-whisker stimulation. The trace is aligned to the beginning of the stimulus, which lasted for 100 msec. Inset: Pipette track, marked with Chicago Sky Blue, in the VPM. Scale bar: 100 µm. (**b**) Analysis of single-unit inter-spike intervals (ISIs). Left: Example histogram of ISIs (expressed as probability density function, PDF) from an identified unit. The bimodal shape of the distribution indicates that the unit displayed bursting. Right: Summary plot of the mean squared coefficient of variation (CV^2) of ISI distribution, a measure of burstiness, for all deprived mice. Each grey line represents the mean CV^2 across all bursting units for each mouse ($n = 12$) between the two hemispheres (Wilcoxon Signed-Rank test; $P \sim 0.03$). (**c**) Center: Example power spectral density (PSD) estimates from the two hemispheres of a control mouse. The traces have been down-sampled to 1Hz for visualization and the scale is the same as in Figure 4. Right: Summary plot of the normalized PSD integral for all control mice ($n = 4$; Wilcoxon Signed-Rank test; n.s. not significant $P = 0.25$).

Supplementary Table 1

Transcripts expression up- or downregulated in ThGrinKO

Downreguated genes	symbol	MeanA	MeanB	absFC	log2FC	p_value	fdr
1449536_at Kcnn1	Kcnn1	7.54898822	3.88791294	1.94165567	0.95728738	7.53E-45	1.73E-40
1418155_at Myot	Myot	6.42527499	3.1388015	2.04704725	1.0335444	2.57E-38	2.95E-34
1426438_at Ddx3y	Ddx3y	10.1952975	7.33332137	1.3902701	0.4753652	4.78E-34	3.66E-30
1437968_at Grin1	Grin1	5.70223008	2.72542899	2.09223212	1.06504292	1.83E-33	1.05E-29
1417210_at Eif2s3y	Eif2s3y	9.94246174	7.06371454	1.40754014	0.49317606	1.20E-26	5.50E-23
1423171_at Gpr88	Gpr88	8.38819309	5.80187604	1.44577255	0.5318406	6.15E-26	2.35E-22
1454608_x_at Ttr	Ttr	4.28703637	2.67767343	1.60103033	0.67900064	7.83E-25	2.56E-21
1450971_at Gadd45b	Gadd45b	7.69195855	5.14822941	1.49409786	0.57927465	8.69E-20	2.49E-16
1449773_s_at Gadd45b	Gadd45b	7.49436452	5.03779678	1.4876274	0.57301323	1.04E-19	2.64E-16
1457651_x_at Rem2	Rem2	9.06932261	6.90093668	1.31421618	0.39420261	6.83E-15	1.57E-11
1448860_at Rem2	Rem2	8.78698782	7.02608892	1.25062292	0.32264686	6.59E-13	1.37E-09
1422861_s_at Pdlim5	Pdlim5	4.51615329	3.38198654	1.33535519	0.41722354	1.85E-12	3.54E-09
1452077_at Ddx3y	Ddx3y	8.29120349	6.64249099	1.24820696	0.31985716	3.36E-12	5.92E-09
1455913_x_at Ttr	Ttr	3.94035815	3.17949207	1.23930429	0.30953046	3.89E-11	6.37E-08
1437385_at Ccbe1	Ccbe1	6.89563886	5.3346867	1.29260428	0.37028067	4.33E-11	6.61E-08
1448468_a_at Kcnab1	Kcnab1	7.72049528	6.87800242	1.12249092	0.16670378	3.60E-10	5.16E-07
1419617_at Kcnn1	Kcnn1	6.6221406	5.04633457	1.31226745	0.39206178	8.43E-10	1.14E-06
1421413_a_at Pdlim5	Pdlim5	5.62166038	4.56723786	1.23086656	0.29967437	1.31E-08	1.59E-05
1450786_x_at Pdlim5	Pdlim5	7.17649662	5.77726214	1.24219681	0.31289377	1.42E-08	1.63E-05
1429783_at Pdlim5	Pdlim5	11.5386961	10.1695102	1.13463636	0.18223	2.39E-08	2.58E-05
1424903_at Kdm5d	Kdm5d	6.0538649	4.55303398	1.32963315	0.41102825	3.05E-08	3.04E-05
1426439_at Ddx3y	Ddx3y	8.18990649	6.79950445	1.20448579	0.26841737	3.18E-08	3.04E-05
1445703_at Ptchd1	Ptchd1	5.98660415	3.90902909	1.53148109	0.61492756	5.37E-08	4.93E-05
1448927_at Kcnn2	Kcnn2	6.6766773	5.61540133	1.18899379	0.24974118	2.06E-07	0.00017503
1434802_s_at Ntf3	Ntf3	6.06511064	4.41765558	1.37292519	0.45725302	3.12E-07	0.00025571
1444062_at 2900056L01Rik	2900056L01	8.31574502	6.74454555	1.23295854	0.30212429	3.90E-07	0.00030367
1443230_at NA	NA	5.12844178	4.00878844	1.27929968	0.35535426	8.11E-07	0.00059523
1447846_x_at Mboat7	Mboat7	4.05478423	3.33717693	1.21503424	0.28099697	8.30E-07	0.00059523
1417150_at Slc6a4	Slc6a4	11.6236077	10.5204844	1.1048548	0.14385679	1.21E-06	0.00083907
1418784_at Grik5	Grik5	5.87500315	4.72059158	1.24454807	0.31562196	2.69E-06	0.00166994
1417943_at Gng4	Gng4	8.95073477	7.93075803	1.12861025	0.17454735	2.84E-06	0.00168053
1442710_at NA	NA	4.53750006	3.33785591	1.35940561	0.44297599	2.90E-06	0.00168053
1448416_at Mgp	Mgp	5.13321054	4.39871658	1.16697915	0.22277879	2.93E-06	0.00168053
1437230_at Kcna1	Kcna1	8.54590484	7.46446621	1.14487823	0.19519416	3.85E-06	0.00215314
1452173_at Hadha	Hadha	5.85039357	4.96377048	1.17861887	0.23709727	4.11E-06	0.00224339
1427445_a_at Ttn	Ttn	6.86030785	5.53684366	1.23902864	0.30920953	7.34E-06	0.00370891
1425845_a_at Shoc2	Shoc2	3.81687962	3.17354052	1.20271967	0.26630042	7.35E-06	0.00370891
1421402_at Mta3	Mta3	6.66245918	6.60656673	1.00846014	0.01215406	8.24E-06	0.00390597
1439908_at Zkscan1	Zkscan1	5.35270982	4.07636039	1.31311006	0.39298784	8.35E-06	0.00390597
1441952_x_at Lynx1	Lynx1	8.17775889	7.12251507	1.14815607	0.19931876	8.74E-06	0.00401003
1429504_at NA	NA	5.74126471	5.03867985	1.13943828	0.18832278	1.18E-05	0.00492219
1423487_at Cript	Cript	6.34476768	5.57434488	1.13820867	0.18676508	1.53E-05	0.00628395
1456330_at Pcgf2	Pcgf2	4.09812883	3.39441211	1.20731623	0.27180361	1.79E-05	0.00698943
1440171_x_at Zfp974	Zfp974	7.41278364	6.49918409	1.14057142	0.18975679	1.80E-05	0.00698943
1442849_at Lrp1	Lrp1	6.08028882	4.08508005	1.48841363	0.5737755	1.97E-05	0.00740628
1445124_at A930009L07Rik	A930009L07	5.97036702	5.17692993	1.15326402	0.20572283	2.12E-05	0.0076037
1456606_a_at NA	NA	9.32200183	8.30481915	1.12248101	0.16669104	2.41E-05	0.00850899
1455785_at Kcna1	Kcna1	10.2347898	9.33483775	1.09640789	0.13278462	2.52E-05	0.0087668
1438516_at NA	NA	3.95591404	3.11649861	1.26934568	0.34408501	2.77E-05	0.00947847
1436239_at Slc5a5	Slc5a5	5.34219175	4.61801885	1.15681462	0.2101577	3.18E-05	0.01040943
1436685_at Fam181a	Fam181a	6.34127755	5.32869041	1.19002551	0.25099251	3.41E-05	0.01100315
1436176_at 1500004A13Rik	1500004A13	4.07761099	3.54659695	1.14972495	0.20128876	3.70E-05	0.01160915
1452324_at Pvt1	Pvt1	7.83812082	6.85738254	1.14301933	0.19284981	3.88E-05	0.01203253
1418991_at Bak1	Bak1	5.49985448	5.05964202	1.08700467	0.12035813	4.54E-05	0.01388252
1456161_at 0610040B10Rik	0610040B10	3.55317541	3.0182217	1.17724136	0.23541013	6.09E-05	0.01789361
1442038_at Rbm26	Rbm26	6.64094609	5.51005211	1.20524198	0.26932282	6.36E-05	0.01845215
1445676_at NA	NA	8.02809574	6.58230113	1.21964881	0.28646579	6.66E-05	0.0186709
1431595_at 2900064B16Rik	2900064B16	5.07355759	4.12477071	1.23002173	0.2986838	6.68E-05	0.0186709
1457633_x_at Cox6a2	Cox6a2	5.84291245	4.73669039	1.23354325	0.3028083	6.99E-05	0.01899632
1422053_at Inhba	Inhba	5.82024017	5.29275312	1.09966213	0.13706033	7.05E-05	0.01899632
1440893_at Riok1	Riok1	5.15948902	4.34630648	1.18709738	0.24743828	7.12E-05	0.01899632
1417416_at Kcna1	Kcna1	8.74008555	7.80753564	1.11944229	0.16278015	9.00E-05	0.02243407
1435610_at Fam19a4	Fam19a4	7.94489286	7.17971554	1.10657488	0.14610108	9.51E-05	0.02319582
1438160_x_at Slco4a1	Slco4a1	4.6114182	3.23788064	1.42420883	0.5101607	0.00010759	0.02543977
1453622_s_at Mllt3	Mllt3	3.98687041	3.44356132	1.15777535	0.21135535	0.00011687	0.02728
1447669_s_at Gng4	Gng4	13.459942	12.3530592	1.08960394	0.12380382	0.00012223	0.02803299
1425248_a_at Tyro3	Tyro3	8.44876419	7.79257159	1.08420745	0.11664083	0.00012667	0.02876393
1423551_at Cdh13	Cdh13	9.33682327	8.49261678	1.09940475	0.13672262	0.00013556	0.03048
1438798_at 4931406P16Rik	4931406P16	5.66224323	4.35380835	1.30052652	0.37909582	0.00017854	0.0375666
1434273_at Fam174b	Fam174b	5.81208325	5.02830209	1.15587392	0.20898404	0.0001981	0.04056652
1433529_at Pamr1	Pamr1	4.17576175	3.55564922	1.17440205	0.2319264	0.00022501	0.04448702

Upregulated genes	symbol	MeanA	MeanB	absFC	log2FC	p_value	fdr
1437434_a_at Wls	Wls	5.40235181	7.28321078	1.34815559	-0.430987	1.42E-09	1.80E-06
1416147_at Hspa4	Hspa4	4.60551987	6.67342531	1.44900587	-0.5350634	2.47E-08	2.58E-05
1440343_at NA	NA	6.42623886	7.94722103	1.23668311	-0.3064759	1.28E-07	0.00011307
1432249_a_at Ercc8	Ercc8	2.77806	4.05656237	1.46021409	-0.5461799	3.97E-07	0.00030367
1456005_a_at Bcl2l11	Bcl2l11	6.84856766	7.52717026	1.09908679	-0.1363053	1.56E-06	0.00105432
1450073_at Kif3b	Kif3b	6.17776734	6.93987525	1.123363	-0.1678242	1.99E-06	0.0013008
1422450_at Ctnnd1	Ctnnd1	2.64171024	3.83285169	1.45089784	-0.5369459	2.18E-06	0.00138925
1439854_at Hrk	Hrk	7.8720574	8.99193322	1.14225961	-0.1918906	4.45E-06	0.00237119
1442845_at C130075A20Rik	C130075A20	4.57130107	5.28231295	1.15553819	-0.2085649	7.44E-06	0.00370891
1436464_at LOC102632463	LOC1026324	6.77388777	8.11140937	1.19745258	-0.2599685	8.22E-06	0.00390597
1435419_at Rgag4	Rgag4	6.40809869	7.67140609	1.19714231	-0.2595947	9.15E-06	0.00411446
1416459_at Arf2	Arf2	5.21629571	6.34235815	1.21587396	-0.2819937	9.78E-06	0.00431362
1415904_at Lpl	Lpl	5.58574253	6.65968408	1.19226478	-0.2537047	1.06E-05	0.0045734
1460092_at NA	NA	3.88683053	4.83230793	1.24325151	-0.3141182	1.15E-05	0.00486383
1420841_at Ptprf	Ptprf	2.91471899	3.86557044	1.32622406	-0.4073245	1.71E-05	0.00689682
1437241_at Klf11	Klf11	7.57800228	8.96193039	1.1826244	-0.2419919	1.86E-05	0.00709599
1432419_a_at Mob2	Mob2	2.90585227	4.0131683	1.38106412	-0.4657803	2.09E-05	0.0076037
1452106_at Npnt	Npnt	6.69419519	8.05816484	1.20375409	-0.2675407	2.09E-05	0.0076037
1448754_at Rbp1	Rbp1	3.72587033	4.40129152	1.18127877	-0.2403495	2.92E-05	0.00983194
1437180_at Snrnp48	Snrnp48	5.19214945	5.95128763	1.14620885	-0.1968699	3.16E-05	0.01040943
1428837_at Klhl14	Klhl14	8.38597368	9.24588842	1.10254203	-0.1408336	3.57E-05	0.01138436
1417658_at Tbrg4	Tbrg4	4.8400634	5.03037465	1.03931999	-0.0556399	4.63E-05	0.01394567
1420644_a_at Sec61a2	Sec61a2	5.27344307	7.17756388	1.36107734	-0.444749	4.68E-05	0.01394567
1439041_at Slc39a10	Slc39a10	5.46585502	6.90026973	1.2624319	-0.3362056	6.59E-05	0.0186709
1441848_at NA	NA	3.38525077	3.41073672	1.00752853	-0.0108207	7.00E-05	0.01899632
1431746_a_at Uba3	Uba3	7.1195885	8.49768782	1.19356446	-0.2552765	7.61E-05	0.01991758
1451117_a_at Tom1l1	Tom1l1	3.69475037	5.27657059	1.42812641	-0.5141237	7.64E-05	0.01991758
1426324_at NA	NA	5.15389264	5.26367732	1.02130131	-0.0304086	7.87E-05	0.02027214
1459713_s_at Ano1	Ano1	3.71899024	4.3117751	1.15939404	-0.213371	8.38E-05	0.02136115
1421922_at Sh3bp5	Sh3bp5	7.56451428	9.14363077	1.20875319	-0.2735197	8.48E-05	0.02136273
1423963_at Wdr26	Wdr26	5.85862482	6.78309211	1.15779595	-0.211381	9.11E-05	0.02246112
1458250_at Fam13c	Fam13c	3.3075387	3.77059462	1.14000015	-0.189034	9.64E-05	0.02326332
1427390_at Bloc1s3	Bloc1s3	6.22527336	7.10084516	1.14064793	-0.1898536	9.78E-05	0.02335698
1433184_at 6720477C19Rik	6720477C19I	5.51703014	6.0290549	1.09280804	-0.12804	0.00011776	0.02728
1460279_a_at Gtf2i	Gtf2i	3.2320343	4.01902814	1.24349798	-0.3144042	0.00013829	0.030792
1455063_at Rmdn2	Rmdn2	5.32167778	5.94149829	1.11647088	-0.1589456	0.00014753	0.03253476
1437587_at NA	NA	3.29915321	3.85176073	1.16749981	-0.2234223	0.00015243	0.03329431
1450008_a_at Ctnnb1	Ctnnb1	8.81597134	10.3547532	1.17454479	-0.2321017	0.0001554	0.03332801
1452899_at Rian	Rian	10.1896717	11.4686886	1.12552091	-0.1705929	0.00015666	0.03332801
1441886_at Terb1	Terb1	3.75517478	3.92497062	1.0452165	-0.0638018	0.00015694	0.03332801
1430143_at 4930426D05Rik	4930426D05	7.09298425	7.81805301	1.10222337	-0.1404166	0.00018346	0.03825091
1435363_at Plekhg1	Plekhg1	9.31361311	10.3246962	1.10855971	-0.1486865	0.00018755	0.03875171
1452373_at Kansl1	Kansl1	7.69374603	8.51137153	1.10627144	-0.1457054	0.00021167	0.04275717
1433989_at Slc6a11	Slc6a11	5.52138403	6.68197348	1.21019901	-0.2752443	0.00021253	0.04275717
1437630_at Lsg1	Lsg1	3.52898241	4.68145844	1.3265746	-0.4077058	0.00021513	0.04290347
1455629_at Drd1	Drd1	4.38025192	5.52076013	1.26037503	-0.3338531	0.00023364	0.04579855
1421491_a_at Vmp1	Vmp1	5.90245515	7.31617029	1.23951307	-0.3097735	0.00023708	0.04608075

Supplementary Table 2

Electrophysiology data analysis at P15

Control	n spikes/pulse (400pA)	Vm	AP width (ms)	AP height (mV)	Series resistance (mOhm)	AP threshold (rheobase) (pA)
1	10	-42.570669	2.7	38.21	12.89004444	20
2	2	-49.761187	1.2	64.16	3.645275	20
3	5	-56.083052	3.4	39.77	9.529096154	20
4	3	-60.294048	4.4	33.37	16.391	40
5	5	-56.19014	4.9	30.95	9.664821111	60
6	9	-57.282396	6	29.56	13.90977692	40
7	4	-35.952788	7.6	28.25	2.145673846	120
8	no data	-60.787719	3	45.5	5.418285714	no data

ThGrin1KO	n spikes/pulse (400pA)	Vm	AP width (ms)	AP height (mV)	Series resistance (mOhm)	AP threshold (rheobase) (pA)
1	25	-51.084716	1.7	48.01	8.843417778	60
2	30	-62.935332	1.7	54.19	12.5483	20
3	12	-36.131114	2	48.31	8.482217273	20
4	28	-57.98335	1.8	44.82	32.44751286	20

IONS	n spikes/pulse (400pA)	Vm	AP width (ms)	AP height (mV)	Series resistance (mOhm)	AP threshold (rheobase) (pA)
1	30	-56.582169	2	51.1	8.335879048	40
2	29	-55.427586	1.8	55.12	2.195732308	40
3	40	-55.767395	2.8	53.18	2.550289167	20
4	42	-56.969757	1.4	58.13	12.9674375	60
5	42	-60.913021	1.8	57.93	9.99093	60

Control+BaCl2	n spikes/pulse (400pA)	Vm	AP width (ms)	AP height (mV)	Series resistance (mOhm)	AP threshold (rheobase) (pA)
1	38	-58.404451	2.4	58.2	10.57525	40
2	35	-59.786331	no data	no data	16.9384	40
3	no data	-29.241934	3.2	58.8	8.161445	no data
4	22	-60.327289	2.8	56.78	9.65895	20
5	18	-56.689655	2.7	58.42	10.6655	40
6	36	-58.790888	1.9	55	7.887045	40

ThGrin1KO+BaCl2	n spikes/pulse (400pA)	Vm	AP width (ms)	AP height (mV)	Series resistance (mOhm)	AP threshold (rheobase) (pA)
1	1	-59.085578	3.5	49.59	2.7568275	20
2	35	-61.448059	2.3	54.28	5.359366667	20
3	31	-49.685535	2.9	59.09	2.664365	20
4	15	-57.210448	3.3	54.84	7.7939	20
5	30	-56.286068	2.17	53.64	5.73344125	20
6	32	-53.44526	3.3	52	7.7788075	40

IONS+BaCl2	n spikes/pulse (400pA)	Vm	AP width (ms)	AP height (mV)	Series resistance (mOhm)	AP threshold (rheobase) (pA)
1	43	-56.443975	3.5	62.61	11.84376471	20
2	32	-55.53124	2.8	61.23	8.17412	20
3	30	-55.53124	2.9	59.71	9.271853333	20
4	30	-59.037062	2.3	59.24	8.92257	20
5	30	-54.863719	1.22	67.89	12.7815	20
6	30	-57.74748	1.96	47.21	11.09503333	20

n spikes/pulse (400pA)		*P-value*
Control vs. ThGrin1KO	**	0.0019
Control vs. IONS	***	<0.001
Control vs. Control+BaCl2	***	<0.001
Control vs. ThGrin1KO+BaCl2	***	<0.001
Control vs. IONS+BaCl2	***	<0.001
**AP height**		
Control vs. IONS	**	0.0036
Control vs. Control+BaCl2	***	0.0008
Control vs. ThGrin1KO+BaCl2	**	0.0045
Control vs. IONS+BaCl2	***	<0.001
**AP width**		
Control vs. ThGrin1KO	*	0.0221
Control vs. IONS	*	0.0219
**AP series**		
ThGrin1KO vs. ThGrin1KO+BaCl2	*	0.0445

Supplementary Table 3

Electrophysiology data analysis at P7

Control	n spikes/pulse (100pA)	Vm	AP width (ms)	AP height (mV)	Series resistance (mOhm)	AP threshold (rheobase) (pA)
1	2.8	-69.66	4.3	41.96	nd	100
2	0.5	-70.09	6.1	37.75	nd	100
3	5.2	-61.78	3.9	49.072	12.38	50
4	0.5	-67.64	2.5	57.28	9.5	100
5	0	-67.34	3.7	42.85	15.78	150
6	0	-62.48	4.5	37.23	nd	200
7	1	-68.18	2.8	50.63	nd	50
8	0	-60.69	4.1	47.64	19.98	150
9	0	-67.05	3.6	13.21	14.6	150

IONS	n spikes/pulse (100pA)	Vm	AP width	AP height	Series resistance (mOhm)	AP threshold (rheobase) (pA)
1	1.8	-70.61	4.8	43.21	14.61	100
2	12.14	-50.63	3.3	54.565	16.67	20
3	14.5	-68.59	3.1	49.13	18.04	50
4	1	-67.75	3.33	43.42	11.64	100
5	0	-69.32	2.9	52.36	14.71	150
6	4	-58.08	4.2	46.41	11.03	100
7	10.75	-57.09	4.4	47.058	9.26	50
8	3.5	-67.02	6.5	32.31	11.82	100
9	8.2	-53.8	2.4	60.64	9.26	100

IONS+Kcna1	n spikes/pulse (100pA)	Vm	AP width	AP height	Series resistance (mOhm)	AP threshold (rheobase) (pA)
1	6.2	-61.31	5.2	12.6	nd	100
2	1	-68.13	6	16.51	19.06	100
3	0	-62.88	5.4	41.9	8.84	150
4	0	-62.63	2	22.46	19.66	200
5	0	-74.44	nd (no AP)	nd (no AP)	7.96	nd (no AP)

n spikes/pulse (100pA)		*P-value*
Control vs. IONS	*	0.0235

AP height		*P-value*
Control vs. IONS+Kcna1	*	0.028
IONS vs. IONS+Kcna1	**	0.0042
